# A Novel Family of Cage-like (CuLi, CuNa, CuK)-phenylsilsesquioxane Complexes with 8-Hydroxyquinoline Ligands: Synthesis, Structure, and Catalytic Activity

**DOI:** 10.3390/molecules27196205

**Published:** 2022-09-21

**Authors:** Alexey N. Bilyachenko, Victor N. Khrustalev, Anna Y. Zueva, Ekaterina M. Titova, Grigorii S. Astakhov, Yan V. Zubavichus, Pavel V. Dorovatovskii, Alexander A. Korlyukov, Lidia S. Shul’pina, Elena S. Shubina, Yuriy N. Kozlov, Nikolay S. Ikonnikov, Dmitri Gelman, Georgiy B. Shul’pin

**Affiliations:** 1 A. N. Nesmeyanov Institute of Organoelement Compounds, Russian Academy of Sciences, Vavilov Str. 28, 119991 Moscow, Russia; 2 Peoples’ Friendship University of Russia (RUDN University), Miklukho-Maklay Str. 6, 117198 Moscow, Russia; 3 Zelinsky Institute of Organic Chemistry, Russian Academy of Sciences (RAS), Leninsky Prospect 47, 119991 Moscow, Russia; 4 Synchrotron Radiation Facility SKIF, Boreskov Institute of Catalysis SB RAS, Nikolskii Prosp. 1, 630559 Koltsovo, Russia; 5 National Research Center “Kurchatov Institute”, Akademika Kurchatova pl. 1, 123182 Moscow, Russia; 6 Pirogov Russian National Research Medical University, Ostrovitianov Str. 1, 117997 Moscow, Russia; 7 Semenov Federal Research Center for Chemical Physics, Russian Academy of Sciences, Ulitsa Kosygina 4, 119991 Moscow, Russia; 8 Chair of Chemistry and Physics, Plekhanov Russian University of Economics, Stremyannyi Pereulok, Dom 36, 117997 Moscow, Russia; 9 Institute of Chemistry, Edmond J. Safra Campus, The Hebrew University of Jerusalem, Jerusalem 91904, Israel

**Keywords:** metallasilsesquioxanes, cage-like compounds, 8-hydroxyquinoline ligands, coordination polymers, half-sandwich units, oxidative catalysis, alkanes, alkyl hydroperoxide

## Abstract

The first examples of metallasilsesquioxane complexes, including ligands of the 8-hydroxyquinoline family **1**–**9**, were synthesized, and their structures were established by single crystal X-ray diffraction using synchrotron radiation. Compounds **1**–**9** tend to form a type of sandwich-like cage of Cu_4_M_2_ nuclearity (M = Li, Na, K). Each complex includes two cisoid pentameric silsesquioxane ligands and two 8-hydroxyquinoline ligands. The latter coordinates the copper ions and corresponding alkaline metal ions (via the deprotonated oxygen site). A characteristic (size) of the alkaline metal ion and a variation of characteristics of nitrogen ligands (8-hydroxyquinoline vs. 5-chloro-8-hydroxyquinoline vs. 5,7-dibromo-8-hydroxyquinoline vs. 5,7-diiodo-8-hydroxyquinoline) are highly influential for the formation of the supramolecular structure of the complexes **3a**, **5**, and **7**–**9**. The Cu_6_Na_2_-based compound **2** exhibits high catalytic activity towards the oxidation of (*i*) hydrocarbons by H_2_O_2_ activated with HNO_3_, and (*ii*) alcohols by *tert*-butyl hydroperoxide. Studies of kinetics and their selectivity has led us to conclude that it is the hydroxyl radicals that play a crucial role in this process.

## 1. Introduction

Nanosized silsesquioxane [RSiO_1.5_]_n_ units bridge a gap between organics and inorganics with their pure inorganic Si-O-Si main chain, surrounded by various organic substituents [1,2,3,4,5]. Silsesquioxanes are often regarded as unique molecular models of silica, both for investigations into surface phenomena [6] and heterogeneous catalysis [7]. These structural features of silsesquioxanes are widely used to design hybrid (organic–inorganic) materials [8,9,10,11]. Another important opportunity provided by silsesquioxanes is their applicability as ligands for a large number of metallacomplexes [12]. In this context, incompletely condensed silsesquioxanes [13] should be mentioned as an especially efficient synthon for the design of different cage-like metallasilisesquioxanes (CLMSs) [14,15,16,17,18,19,20,21,22,23,24]. As a result, CLMSs offer an impressively wide scope of applications. These include the investigation of flame-retardant properties [25,26,27,28,29,30] and the development of approaches to anode [31,32] and ceramic [33] materials. Recent results revealed intriguing magnetic effects [34,35] (including the observation of spin glass [36] and single-molecule magnet [37] behaviors) and photophysical properties [38,39,40,41,42,43,44] of CLMSs. CLMSs are widely discussed as homo- and heterogeneous catalysts [45,46,47,48,49], with the most recent papers reporting the impressive activity of metallasilsesquioxanes in (*i*) Chan–Evans–Lam coupling [50], (*ii*) the hydroboration of ketones [51], (*iii*) CO_2_ valorization [52], (*iv*) the synthesis of bio-derived ethers [53], and (*v*) oxidative amidation [54].

Additionally, it is extremely well known that transition metal complexes efficiently catalyze the oxidations of hydrocarbons and alcohols involving the functionalization of C–H bonds [55,56,57,58]. Copper derivatives bearing various nitrogen-containing ligands are among the most active catalysts in the oxidation of organic compounds with peroxides [59,60,61,62,63,64,65,66,67,68,69], and this applies to the full extent of several Cu-based sesquioxane complexes [70,71,72,73,74]. Interested in further studying functional cage-like metallasilsesquioxanes, we have synthesized a series of novel copper silsesquioxanes via complexation with ligands of the 8-hydroxyquinoline family. Recent publications by us [50,75,76,77,78,79] and others [37,40,51,80,81] showed that the self-assembly of CLMSs in the presence of various (N,N-, P,P-, O,O-) organic ligands could be regarded as an efficient and facile approach to new types of mixed-ligand metallasilsesquioxane complexes.

To the best of our knowledge, such a popular bidentate ligand as 8-hydroxyquinoline has never been applied for the design of CLMS derivatives, despite an enormous number of other 8-hydroxyquinoline complexes and their multiple applications [82,83,84,85,86]. Here, we present our results on the synthesis of the very first examples of heteroligand silsesquioxane/8-hydroxyquinoline metallacomplexes and their evaluation towards the oxidative functionalization of hydrocarbons.

## 2. Results and Discussion

### 2.1. Synthesis and Structure

**Scheme 1:**
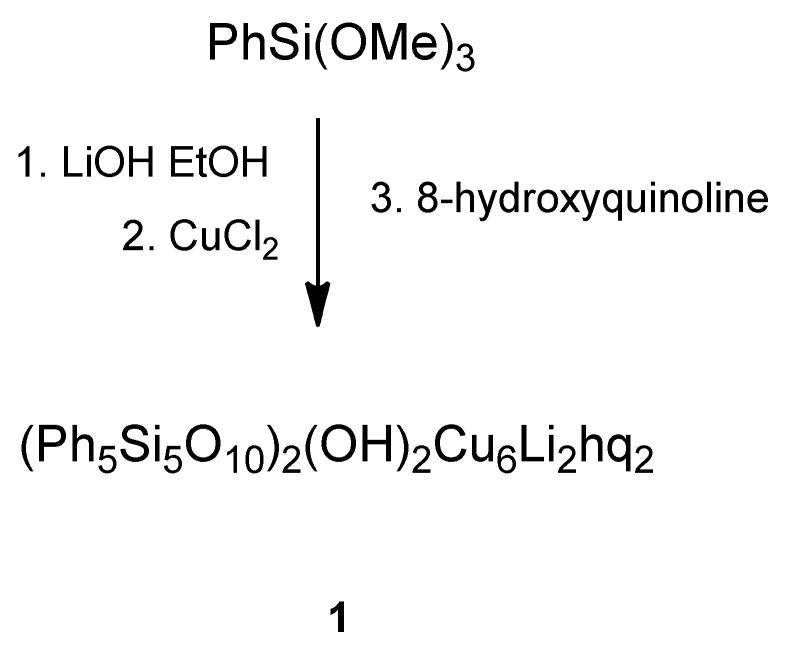
General scheme of the synthesis of CuLi-silsesquioxane/8-hydroxyquinoline complex **1**. Solvated molecules are omitted for clarity.

For a synthesis method, a convenient “siloxanolate route” was chosen. This approach implies the in situ formation of active lithium siloxanolate [PhSi(O)OLi]_n_ species via the interaction of PhSi(OMe)_3_ with LiOH in an ethanol solution. Later on, these species interact with CuCl_2_, and the copper–lithium silsesquioxane formed in situ is treated (Scheme 1) with 8-hydroxyquinoline (hq). The use of lithium hydroxide for the CLMS design is in accordance with recent publications [30,87]. At the same time, classical approaches rely on more complicated reactions with either BuLi/LiN(SiMe_3_)_2_ (for metalation synthesis [88,89,90,91]) or LiCl (for transmetalation synthesis [92]).

As a result, the very first example of a CLMS/8-hydroxyquinoline complex, compound **1** of [(Ph_5_Si_5_O_10_)_2_(OH)_2_Cu_6_Li_2_hq_2_(EtOH)_2_]·EtOH composition, was isolated in a 33% yield.

**Figure 1:**
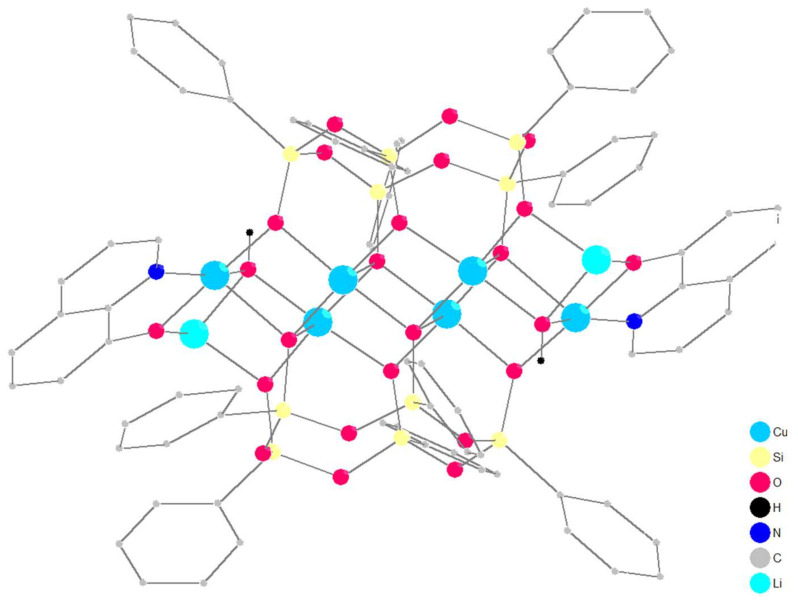

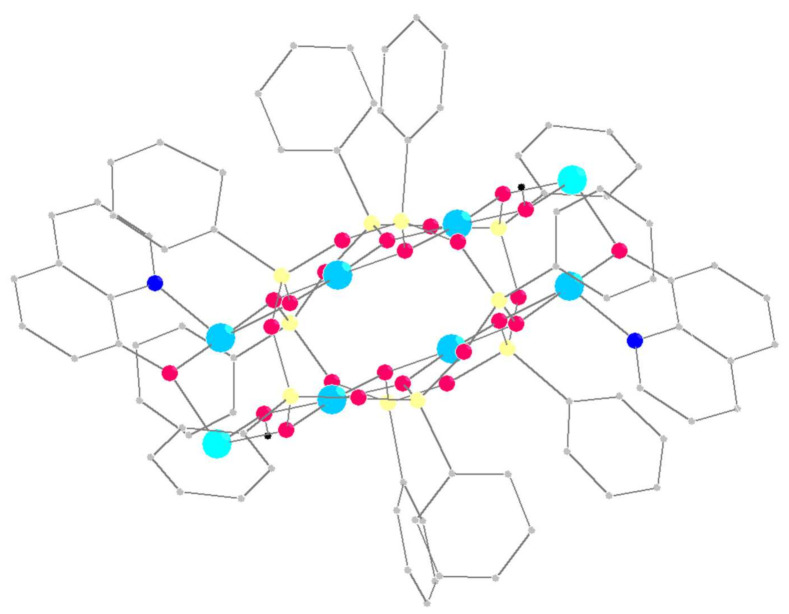
Two projections of the molecular structure of **1**.

**Figure 2:**
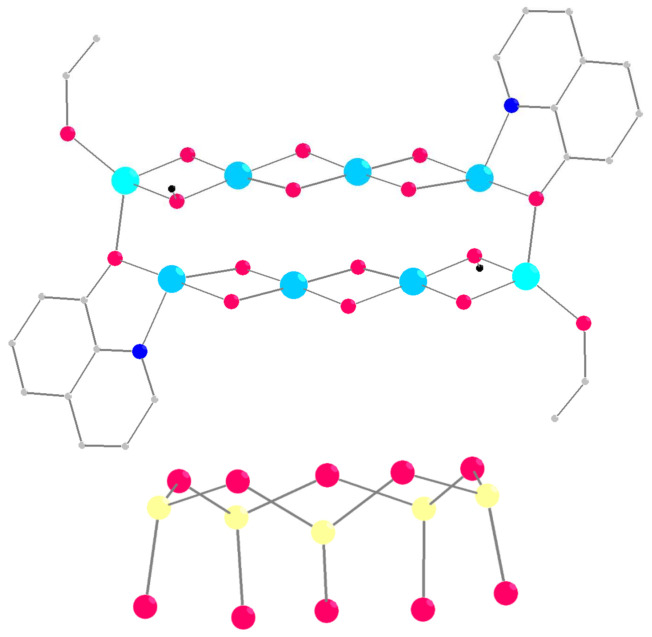
**Top**. The metal-containing central core of **1**. **Bottom**. The silsesquioxane ligand of **1**. Phenyl groups at the silicon atoms are not shown. The color code is the same as in Figure 1.

Compound **1** represents the skewed sandwich-like cage structure, as established by an X-ray diffraction study (Figure 1). The central core of complex **1** includes two almost linear Cu…Cu…Cu…Li fragments (Figure 2, top), coordinated by two cyclic silsesquioxane [Ph_5_Si_5_O_10_] ligands (Figure 2, bottom). Obviously, the Cu^II^_6_ nuclearity (giving 12 positive charges) could not be compensated by two pentameric silsesquioxane ligands (giving 10 negative charges). The electroneutrality of complex **1** is reached by the presence of two hydroxyl groups. Additionally, two opposite copper ions are coordinated by 8-hydroxyquinolinolate ligands. In turn, the hydroxyl groups of the pristine 8-hydroxyquinoline molecules are metallated by lithium ions, forming OLi units. It is worth nothing that, despite the enormous popularity of 8-hydroxyquinoline ligands, cage **1**, to the best of our knowledge, is only the second example of dual M-O-M’ (copper/alkali metal) complexation (M = Cu and M’ = an alkali metal), with the first one being reported for a Cu^I^K 8-hydroxyquinoline compound [93].

The molecular structure of “complex **1** type” is easily reproduced. Additional runs of this reaction produced complex **1a** of the [(Ph_5_Si_5_O_10_)_2_(OH)_2_Cu_6_Li_2_hq_2_(EtOH)_1.6_(H_2_O)_0.4_]·EtOH_sq_ composition in a 39% yield. Complex **1a** is a close analog of compound **1** (the only exception being a partial replacement of ethanol molecules coordinating lithium centers by water molecules). This difference causes negligible changes in cage structures (e.g., the intramolecular Li…Li distance is equal to 10.3512(2) Å and 10.3347(1) Å for **1** and **1a**, respectively).

**Scheme 2:**
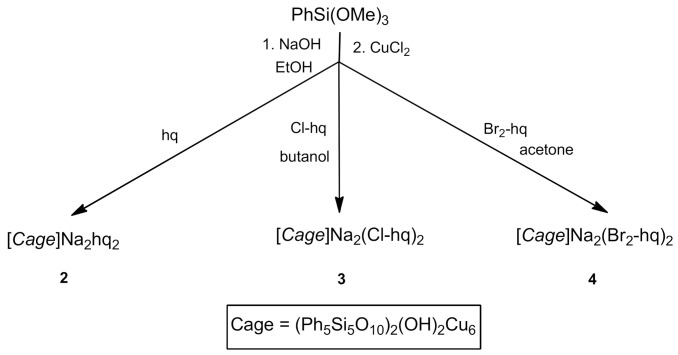
General scheme of the synthesis of CuNa-silsesquioxane complexes with ligands of the 8-hydroxyquinoline family **2**–**4**. Solvated molecules are omitted for clarity.

**Figure 3:**
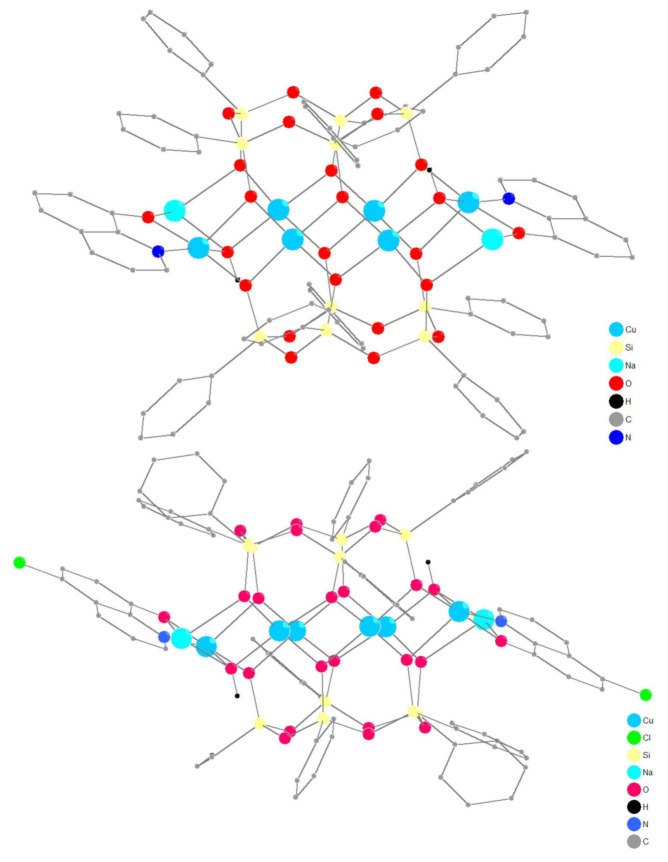

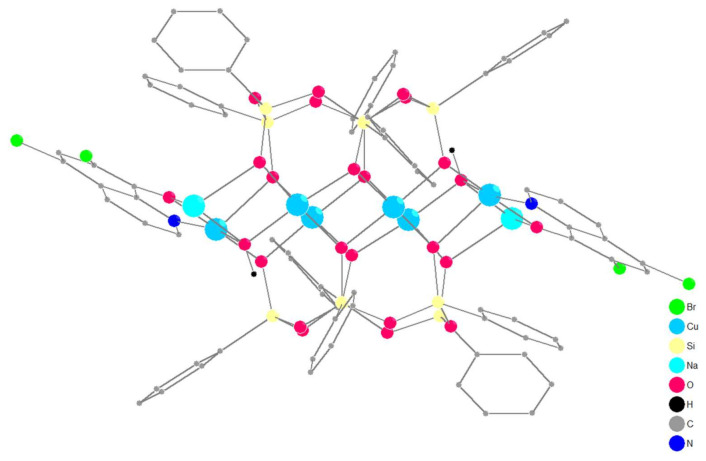
**Top**. The molecular structure of **2**. **Middle**. The molecular structure of **3**. **Bottom**. The molecular structure of **4**.

Further exploration of this reaction, namely a shift to sodium-based complexes (via the replacement of LiOH reagent by NaOH in Scheme 2), allowed us to isolate a series of CuNa cage compounds **2**–**4**. These compounds include complex **2** of the [(Ph_5_Si_5_O_10_)_2_(OH)_2_Cu_6_Na_2_hq_2_(EtOH)_2_(H_2_O)]·EtOH composition (32% yield, Figure 3, top). Additionally, implementation of 5-chloro-8-hydroxyquinoline (Cl-hq) allowed us to isolate complex **3** of the [(Ph_5_Si_5_O_10_)_2_(OH)_2_Cu_6_Na_2_(Cl-hq)_2_(*n*-BuOH)_4_] composition (20% yield, Figure 3, middle). Finally, the use of 5,7-dibromo-8-hydroxyquinoline (Br_2_-hq) allowed us to isolate complex **4** of the [(Ph_5_Si_5_O_10_)_2_(OH)_2_Cu_6_Na_2_(Br_2_-hq)_2_(EtOH)_2_(Me_2_CO)(H_2_O)]·2Me_2_CO EtOH composition (44% yield, Figure 3 bottom). Compounds **2**–**4** represent the same type of molecular architecture as **1**–**1a**. Differences in solvate systems and the nature of 8-hydroxyquinolinolate ligands cause negligible changes in the structural features of their cages (e.g., the intramolecular Na…Na distance is equal to 11.4133(4) Å, 11.4487(2) Å, and 11.4005(6) Å for **2**, **3**, and **4**, respectively). Nevertheless, we would like to mention, once again, the acetone-solvated compound **4**. We have recently reported the use of acetone as the ligand of choice as a very efficient method of isolating crystalline cage metallasilsesquioxanes (12 complexes) [94]. The smooth formation of complex **4** (in a higher yield than for compounds **1**–**3**) supports the conclusion concerning the acetone potential in the CLMS design. In turn, a change in the solvate system used for the synthesis of complex **3**, namely the application of an EtOH solution (Supplementary Scheme S1), results in the isolation of compound **3a** of the [(Ph_5_Si_5_O_10_)_2_(OH)_2_Cu_6_Na_2_(Cl-hq)_2_(EtOH)_4_] composition in a 26% yield. In terms of molecular structure, compounds **3** and **3a** are very close analogs (the intramolecular Na…Na distances are equal to 11.4487(2) Å and 11.4747(4) Å for **3** and **3a**, respectively). At the same time, unlike the discrete structure of **3**, compound **3a** exhibits T-stacking interactions between the phenyl groups of neighboring cage units (Supplementary Figure S1). This points to a strong influence of solvate ligands on the supramolecular assembly of CLMSs, as will be discussed in more detail below.

**Scheme 3:**
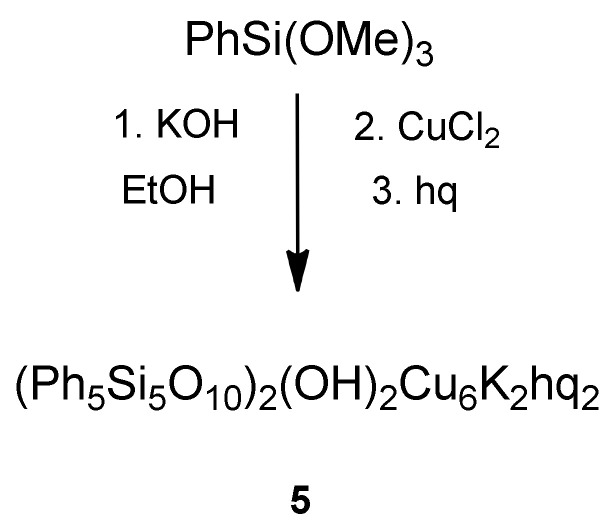
General scheme of the synthesis of CuK-silsesquioxane/8-hydroxyquinoline complex **5**. Solvated molecules are omitted for clarity.

**Figure 4:**
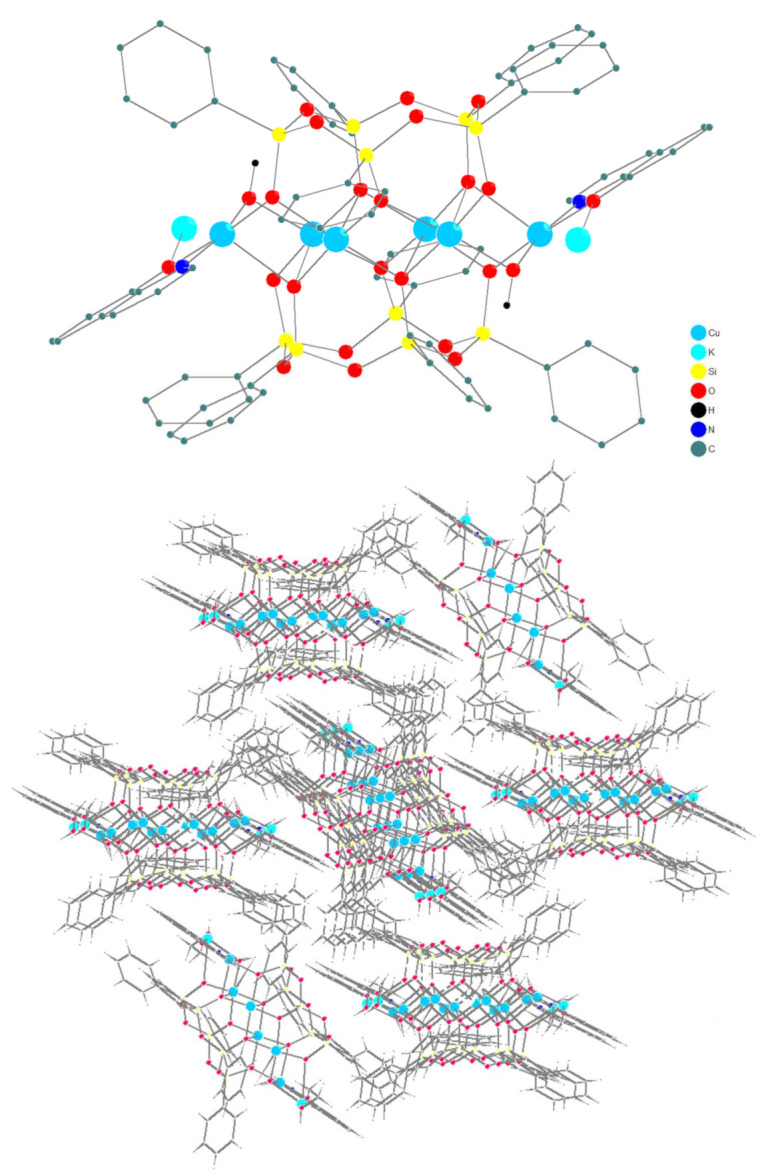
The molecular structure (**top**) and crystal packing (**bottom**) of **5**.

**Figure 5:**
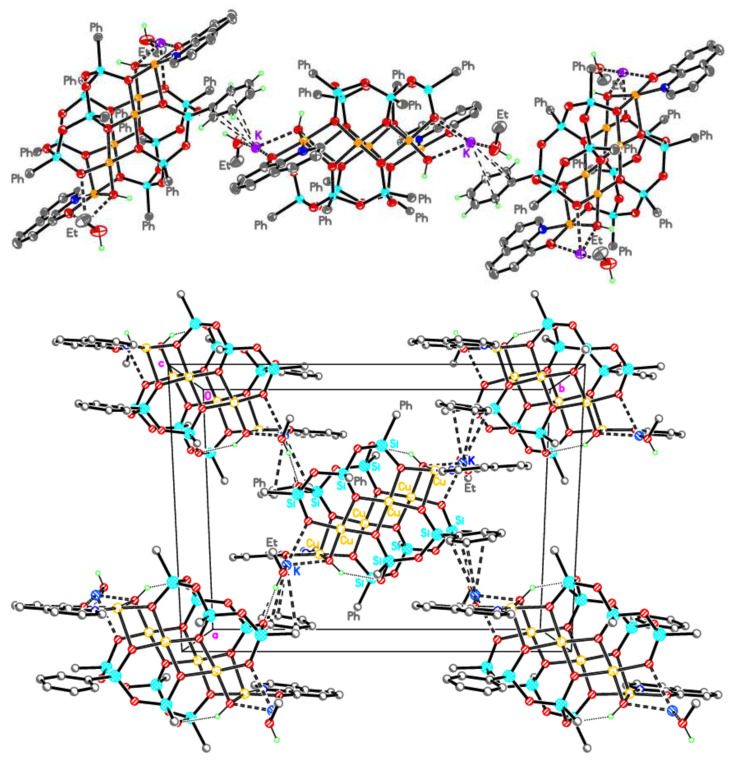
**Top**. Principle of the metallocene (potassium–phenyl) linkage observed in **5**. **Bottom**. A fragment of the 2D coordination polymer structure of **5**.

A further investigation of such self-assembly reaction conditions (with KOH as a starting reagent, Scheme 3) allowed us to isolate the 8-hydroxyquinolinolate-containing complex **5**. Complex **5** of the [(Ph_5_Si_5_O_10_)_2_(OH)_2_Cu_6_K_2_(hq)_2_(EtOH)_2_] composition (37% yield, Figure 4, top) represents the same fashion of molecular architecture as discussed for complexes **1**–**4**. At the same time, the strong individuality of complex **5** is revealed by its unusual supramolecular structure (Figure 4, bottom). The inter-cage connections are realized via the metallocene K·π-Ph joints between the potassium center of one cage and the phenyl group of the silicon atom, which belongs to a neighboring cage (Figure 5, top). Only two similar observations could be cited in this context:(*i*) Na·π-Ph joints in discrete Mn,Na CLMS [95] and (*ii*) Cs(Rb)·π-Ph joints in CuCs(Rb) CLMS coordination polymers [96]. Interestingly, each cage complex in the supramolecular structure of **5** interacts with neighbors via both two potassium centers and two phenyl groups, thus forming an unusual 2D coordination polymer structure (Figure 5, bottom).

**Scheme 4:**
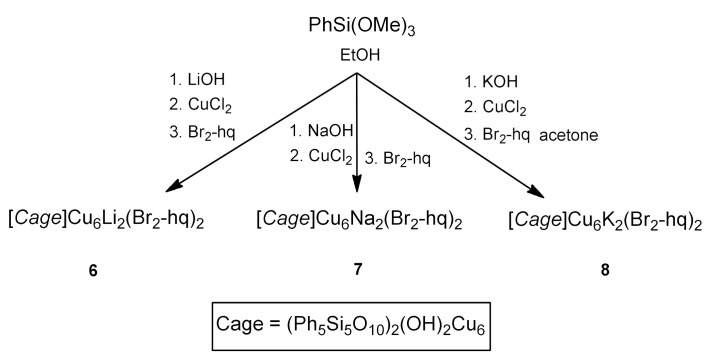
General scheme of the synthesis of CuLi- (**6**), CuNa- (**7**), and CuK-based (**8**) silsesquioxane complexes with 5,7-dibromo-8-hydroxyquinoline ligands. Solvated molecules are omitted for clarity.

Nevertheless, a general conclusion that could be derived from the comparison of complexes **1**–**5** agrees with the results of aforementioned publications [97,98,99] that a large alkali metal ion could be easily involved in inter-cage connections. Indeed, the only coordination polymer among complexes **1**–**5**, compound **5**, includes potassium ions (unlike lithium- or sodium-based cages **1**–**4**). To make a correct conclusion regarding the role played by the alkali metal ion, a series of additional experiments with one type of ligand, namely 5,7-dibromo-8-hydroxyquinoline, was performed (Scheme 4). These experiments let us isolate three compounds, including the reference alkali metal ions: lithium-complex [(Ph_5_Si_5_O_10_)_2_(OH)_2_Cu_6_Li_2_(Br_2_-hq)_2_(EtOH)_2_]·4 EtOH **6**, sodium-complex [(Ph_5_Si_5_O_10_)_2_(OH)_2_Cu_6_Na_2_(Br_2_-hq)_2_(EtOH)_2_]·4 EtOH **7**, and potassium-complex [(Ph_5_Si_5_O_10_)_2_(OH)_2_Cu_6_K_2_(Br_2_-hq)_2_(Me_2_CO)_4_]·2Me_2_CO **8** in 18%, 27%, and 39% yields, respectively.

**Figure 6:**
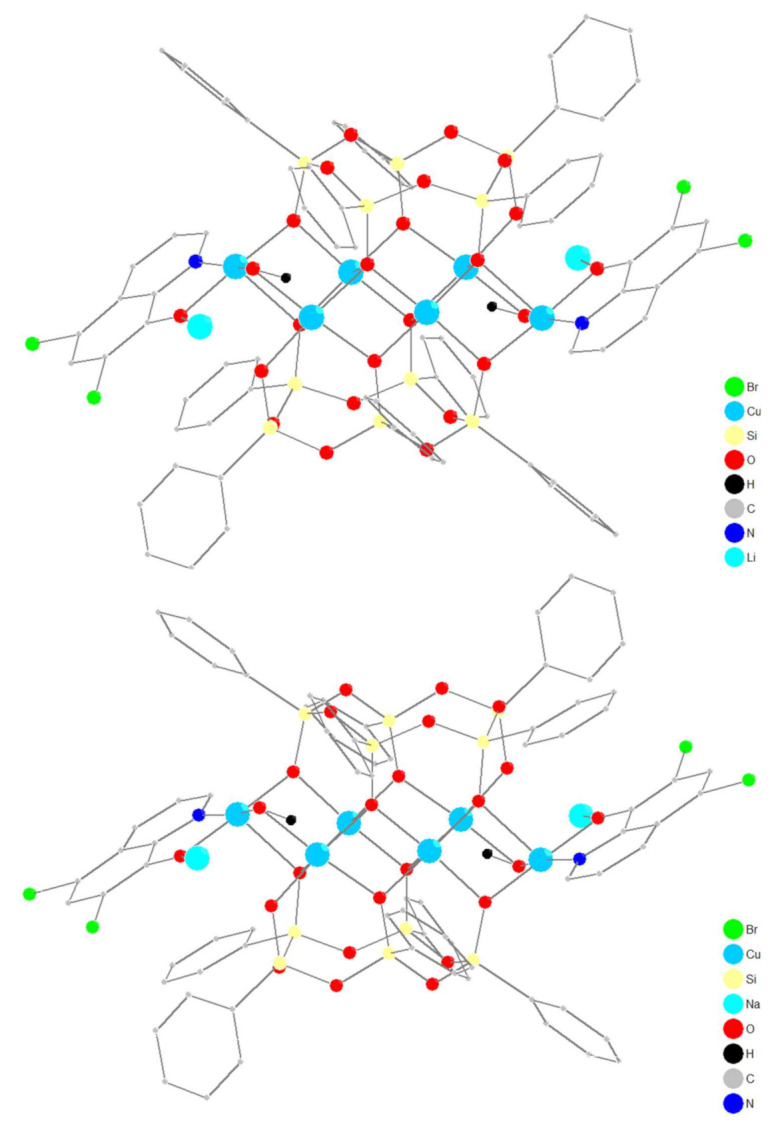

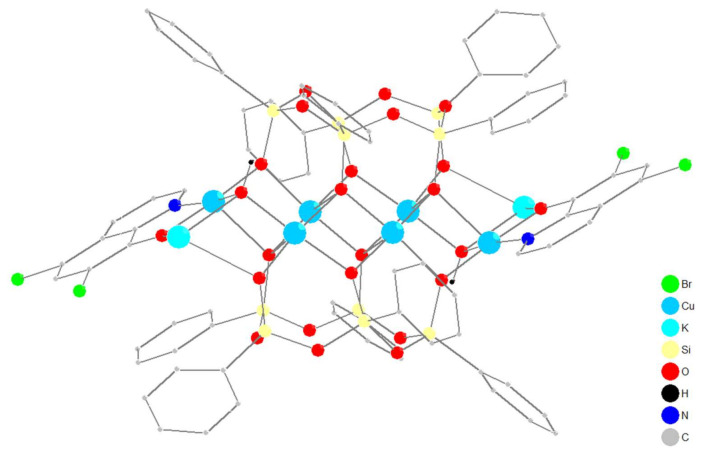
**Top**. The molecular structure of **6**. Middle. The molecular structure of **7**. **Bottom**. The molecular structure of **8**.

**Figure 7:**
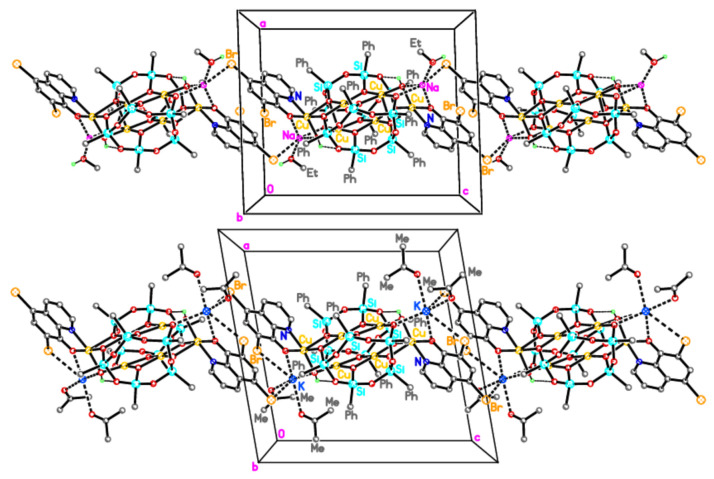
**Top**. A fragment of the 1D coordination polymer structure of **7**. **Bottom**. A fragment of the 1D coordination polymer structure of **8**.

All these complexes (Figure 6) belong to a general type of molecular architecture discussed for **1**–**5**. What is important is that compounds **6**-**8** exhibit profound differences in the context of their supramolecular geometry. According to formal expectations, complex **6**, containing smaller (lithium) ions, exhibits no supramolecular structure. In turn, both compounds **7**–**8**, containing larger sodium or potassium ions, respectively, pack in the crystal in a way that forms a ladder-like motif (Figure 7, top and bottom, respectively). Unlike the “metallocene-connected” 2D coordination polymer **6**, 1D coordination polymers **7**–**8** are assembled via almost rectangular units, formed by two additional contacts between the bromide center of Br_2_-hq ligand of one cage and sodium (or potassium) center of the neighboring cage. Distances Na…Br (in **7**) and K…Br (in **8**) are equal to 3.317(2) and 3.3816(10) Å, respectively, which points to their coordination/ionic character. Here, it is especially interesting to compare the “supramolecular” behaviors of close analogs, namely compounds **4** and **7**,Cu_6_Na_2_-based complexes with 5,7-dibromo-8-hydroxyquinoline ligands. While compound **4** crystallizes in an island-like structure, compound **7** forms the aforementioned 1D coordination polymer. As the only difference between these two complexes is a set of solvate ligands (acetone/ethanol/water in the case of **4**, and ethanol only in the case of **7**), the role played by the solvate molecules in the formation/cleavage of the inter-cage association could be concluded. This observation is in perfect accordance with several earlier reports [72,97,98,100,101], and will definitely yield more confirmations in future research.

**Scheme 5:**
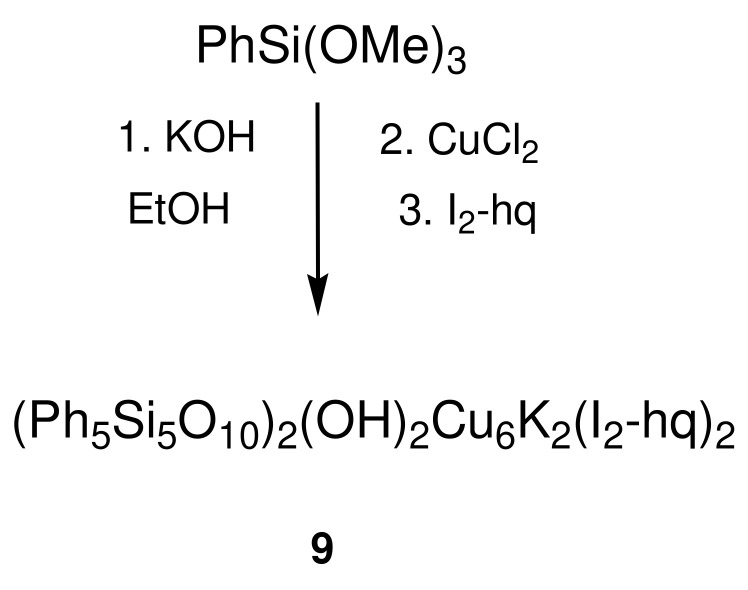
General scheme of the synthesis of CuK-silsesquioxane/5,7-diiodo-8-hydroxyquinoline ligand complex **9**. Solvated molecules are omitted for clarity.

**Figure 8:**
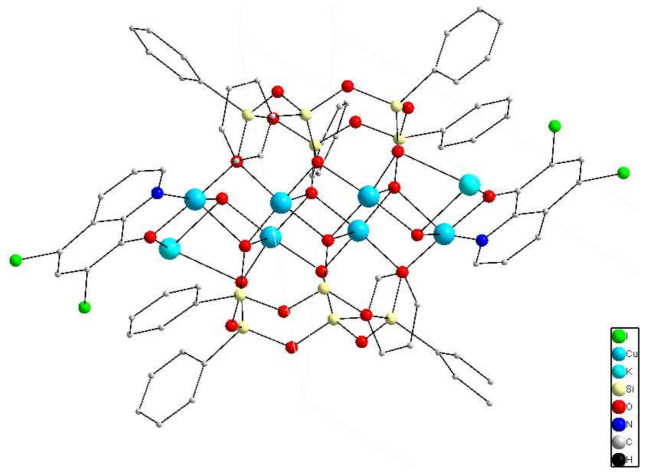
The molecular structure of **9**.

**Figure 9:**
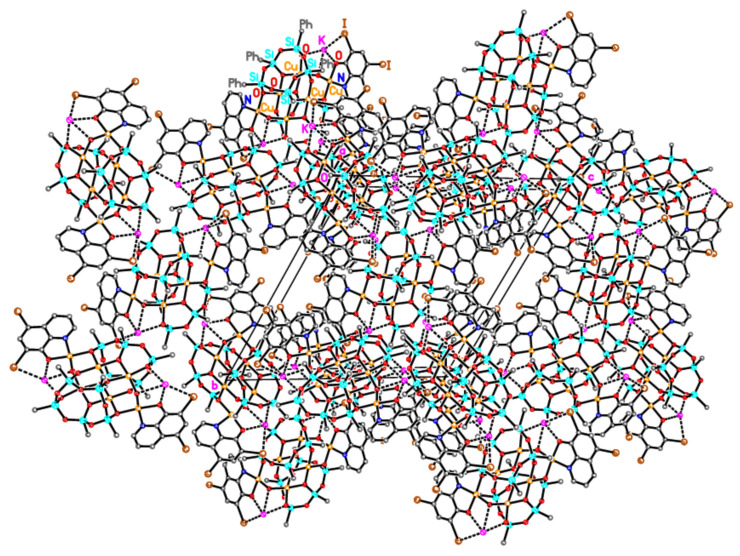
The 3D coordination polymer structure of **9**.

Considering the high efficiency of large-sized alkali metal ions, it was interesting to study the same effect for modified 8-hydroxyquinoline ligands bearing extra substituents. In this line of thought, the self-assembly synthesis of CuK-phenylsilsesquioxane with 5,7-diiodo-8-hydroxyquinoline (I_2_-hq) ligands has been performed (Scheme 5). This reaction smoothly produced CLMS **9** of the [(Ph_5_Si_5_O_10_)_2_(OH)_2_Cu_6_K_2_(I_2_-hq)_2_]·⅙MeOH composition in a 33% yield. The type of molecular structure of complex **9** (Figure 8) is similar to those of **1**–**8**, but its supramolecular architecture is quite different compared to compounds **5**, **7**, and **8.** To our surprise, no iodide centers get involved in the supramolecular interactions (K…I interactions are normal non-valence intramolecular ones). Nevertheless, the molecules in **9** pack into an unprecedented 3D structure via the crown ether-like contacts of the potassium ion of one cage and two oxygen atoms of the silsesquioxane ligand of the neighboring cage (Figure 9). Thus, in the crystal, compound **9** forms the unique “solvent-free” 3D framework via potassium ions, binding no solvate methanol molecules.

In the context of functional properties, a representative compound **2** (Cu_6_Na_2_-based complex) has been tested as a homogeneous catalyst towards the oxidative functionalization of hydrocarbons (including cyclohexane and *n*-heptane) and alcohols.

### 2.2. Catalyzed Oxidation of Cyclohexane

**Figure 10:**
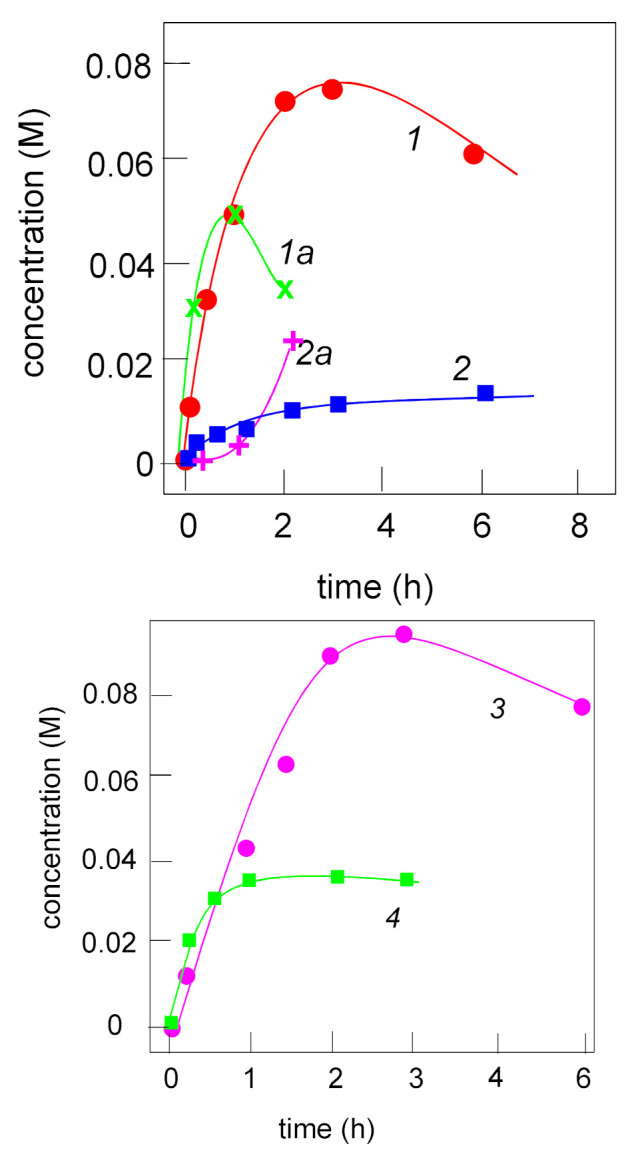
Oxidation of cyclohexane (0.46 M) with hydrogen peroxide (50% aqueous; 2.0 M) catalyzed by complex **2** (concentration 5 × 10^−4^ M) in the presence of nitric acid (0.05 M) in acetonitrile at 50 °C. Concentrations of cyclohexanol (curve *1*), cyclohexanone (curve *2*), and sum cyclohexanol + cyclohexanone (curve *3*) were determined after the reduction of the aliquots with solid PPh_3_. Concentrations of cyclohexanol (curve *1a*) and cyclohexanone (curve *2a*) in the absence of nitric acid in the analogous experiment. For the comparison, the accumulation of cyclohexanol + cyclohexanone in the oxidation of cyclohexane with *tert*-BuOOH under the same conditions is represented by curve *4*.

**Figure 11:**
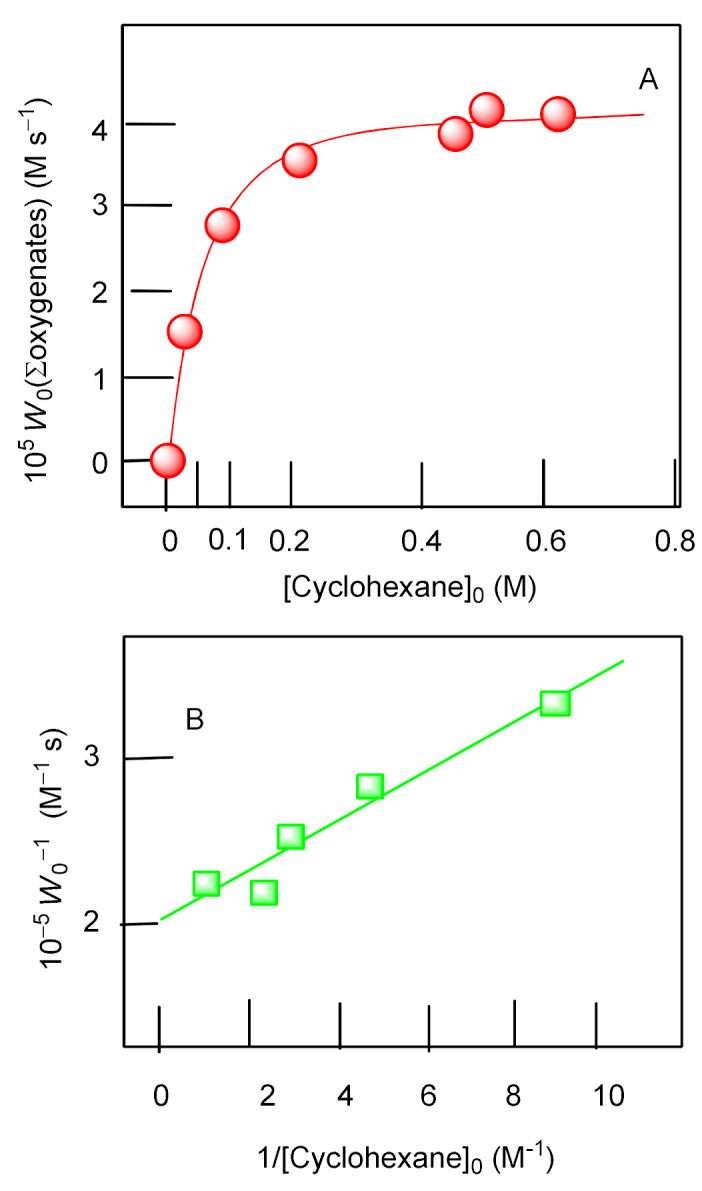
Dependence of the initial rate of oxygenate (sum cyclohexanol + cyclohexanone) formation *W*_0_ for complex **2** on the initial concentration of cyclohexane (graph A). Linearization of the curve from graph A in coordinates *W*_0_^−1^ − 1/[C_6_H_12_] is shown in graph B. Conditions. Concentrations: [1] = 5 × 10^−4^ M, [H_2_O_2_]_0_ = 2.0 M, [HNO_3_] = 0.05 M, in acetonitrile at 50 °C. Concentrations of cyclohexanone and cyclohexanol were determined after the reduction of the aliquots with solid PPh_3_.

Complex **2** was studied more carefully in the oxidation of hydrocarbons with cyclohexane as a case example. Typical oxidation kinetics curves of cyclohexane are presented in Figure 10. It should be noted that under the studied conditions, nitric acid additives practically do not affect the rate of formation of cyclohexyl hydroperoxide within an hour. However, these additives have a significant effect on the subsequent course of the process. The catalyst deactivation observed during the reaction is apparently due to the destruction of organic ligands bound to copper ions and its hydrolysis in the presence of water traces. The striking similarity of the initial oxidation rates with hydrogen peroxide and *tert*-butyl hydroperoxide is also worth noting. It indicates a similar reactivity of these peroxides in their catalytic conversion under the action of **2**. Initial reaction rates were found at various initial cyclohexane concentrations, presented below in Figure 10 and Figure 11.

We found that the rate of cyclohexyl hydroperoxide formation at a concentration of cyclohexane of 0.05 M decreases by a factor of 4.3 with the addition of linear alkane *n*-heptane at a concentration of 0.4 M. At the same time, the total yield of alkyl hydroperoxides generated from cyclohexane and *n*-heptane practically coincides with the yield of cyclohexyl hydroperoxide at a concentration of 0.46 M, in the absence of *n*-heptane additives. These data, as well as the mode of dependence of the initial rate of cyclohexyl hydroperoxide formation on the cyclohexane concentration (Figure 11), indicate that the stimulation of alkane oxidation is due to the same intermediate species, which decomposes hydrogen peroxide.

Taking into account the data presented above, as well as data on the selectivity of the oxidation of linear alkanes (the ratios of relative reactivities are C-1:C-2:C-3:C-4 = 1.0:2.0:2.4:2.5 for the *n*-heptane oxidation and 1:2:3 = 1.0:5.6:14.5 for the methylcyclohexane oxidation), indicates that it is hydroxyl radicals that act as the active species. The kinetic scheme, which describes the obtained results, is shown below:

**2**→ Z (1)

Z → Y(2)

H_2_O_2_ + Z →…→ HO^•^ (3)

HO^•^ + CH3CN →…→ oxidation products (4)

HO^•^ + C_6_H_12_ →…→ ROOH (5)

HO^•^ +n-C_7_H_16_ →…→ rOOH (6)

Here, the initial stage (Equation (1)) includes the transformation of the pristine complex **2** in the presence of water and 0.05 M HNO_3_ in the intermediate state Z, which genuinely manifests catalytic activity in the decomposition of H_2_O_2_ to generate hydroxyl radicals at a rate of *W*_3_ (for reaction 3).

The next step (Equation (2)) is the transformation of Z into a catalytically inactive form, Y.

Reactions (Equation (3)) are the sequence of transformations leading to the formation of HO^•^ radicals. The limiting stage of these reactions is the interaction of H_2_O_2_ with species Z.

Equations (4)–(6) describe the sequence of the transformations leading to the formation of oxidation products of acetonitrile (Equation (4)), cyclohexyl hydroperoxide (Equation (5)), and *n*-heptane hydroperoxides (Equation (6)), with the limiting stages being the HO^•^﮲ interactions with CH_3_CN, C_6_H_12_, and *n*-C_7_H_16_, characterized by the rate constants *k*_4_, *k*_5_, and *k*_6_, respectively.

From the proposed kinetic scheme, within the framework of the quasi-stationary approximation, it is easy to obtain expressions for the rate of formation of ROOH (Equation (7)) and the rate of formation of the sum of peroxides ROOH and rOOH (Equation (8)).

d [ROOH]/d*t* = *W*_3_ • *k*_5_ [C_6_H_12_]/(*k*_4_ [CH_3_CN] + *k*_5_ [C_6_H_12_] + *k*_6_ [*n*-C_7_H_16_]) (7)

d ([ROOH] + [rOOH])/d*t* = *W*_3_ • (*k*_5_ [C_6_H_12_] + *k*_6_ [*n*-C_7_H_16_])/(*k*_4_ [CH_3_CN] + *k*_5_ [C_6_H_12_] + *k*_6_ [*n*-C_7_H_16_]) (8)

Let us employ Equations (7) and (8) to analyze the observed effect of the *n*-heptane additive. The proximity of the formation rates of ROOH at [C_6_H_12_] = 0.46 M and [*n*-C_7_H_16_] = 0 and the sums of ROOH and rOOH at [C_6_H_12_] = 0.05 M and [*n*-C_7_H_16_] = 0.4 M allows us to obtain the ratio 0.46*k*_5_/(*k*_4_ [CH_3_CN] + 0.46*k*_5_) ≈ (0.05*k*_5_ + 0.4*k*_6_)/(*k*_4_ [CH_3_CN] + 0.05*k*_5_ + 0.4*k*_6_), which is valid at *k*_5_ ≈ *k*_6_. The coincidence of rate constants of the interaction of HO^•^ radicals with cyclohexane and *n*-heptane is consistent with published data [102,103,104]. The decrease in the rate of ROOH formation at [C_6_H_12_] = 0.05 M from the addition of 0.4 M *n*-C_7_H_16_ by a factor of 4.3 allows us to make another estimate of the ratio of rate constants. Using (Equation (7)) for these conditions and the relation *k*_5_ ≈ *k*_6_ obtained above, we have:

(*k*_4_[CH_3_CN]/0,05 *k*_5_ + 1 + 8)/(*k*_4_[CH_3_CN]/0.05*k*_5_ + 1) = 4.3.

*k*_4_[CH_3_CN]/*k*_5_ = 0.086 M.

From the last relation, it follows that *k*_4_ [CH_3_CN]/*k*_5_ = 0.071 M.

An analysis of the experimental dependence of the initial rate of ROOH formation on the concentration of C_6_H_12_ (Figure 11A) in the coordinates corresponding to the linear anamorphosis of Equation (7), [C_6_H_12_]/(d [ROOH]/d*t*)_0_—ordinate, [C_6_H_12_]—abscissa (its results are presented in Figure 11B), showed the correspondence of the experimental data of the proposed kinetic model; a satisfactory linear relationship is observed in the proposed coordinates. Further, from the ratio of the magnitude of the segment of this dependence to the tangent of the angle of inclination, it follows:

*k*_4_ [CH_3_CN]/*k*_5_ = 0.086 M.

This value of the ratio of rate constants, as well as the value obtained above from the data on the effect of *n*-heptane additives on the rate of ROOH formation at [C_6_H_12_] = 0.05 M, are close to the value characteristic of reactions involving hydroxyl radicals. Thus, in addition to the regioselectivity data for the oxidation of linear alkanes, which indicate the induction of the oxidation reaction by hydroxyl radicals, we also have kinetic evidence for this.

### 2.3. Catalyzed Oxidation of Alcohols

**Figure 12:**
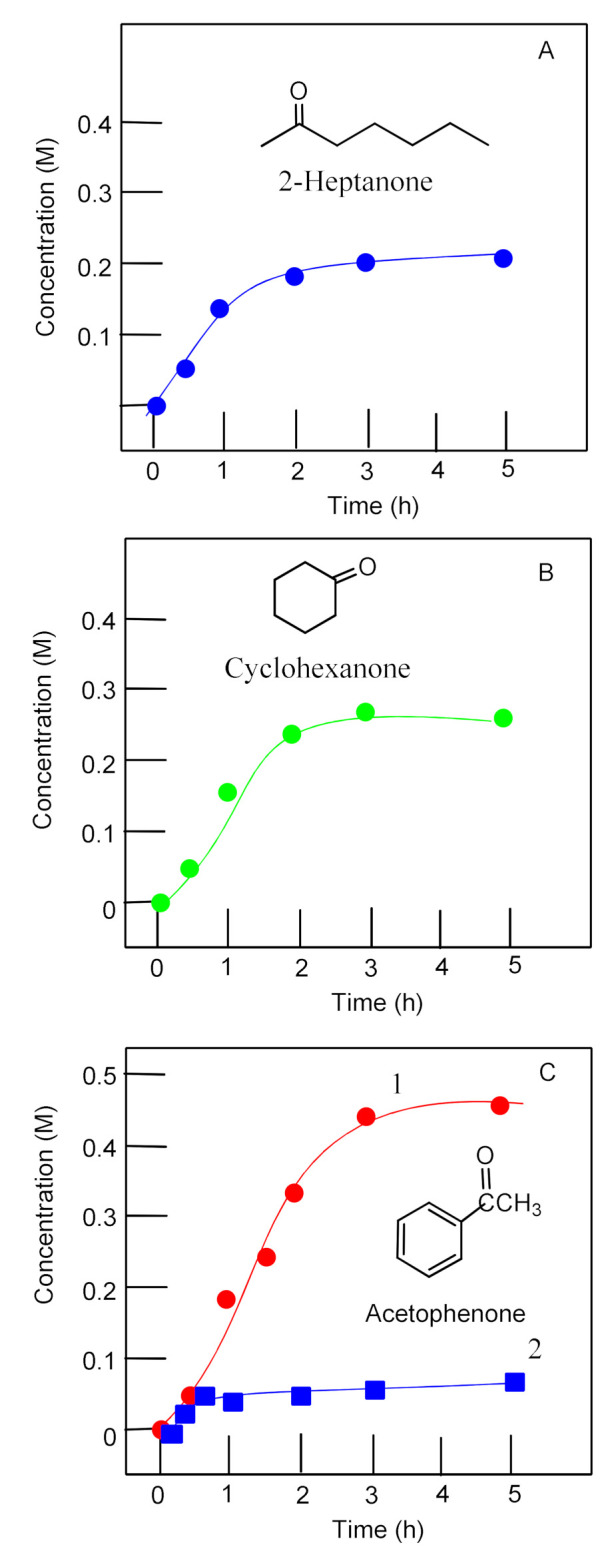
Accumulation of 2-heptanone (graph **A**), cyclohexanone (graph **B**), and acetophenone (graph **C**), (curve 1), in the oxidation of 2-heptanol (0.42 M), cyclohexanol (0.5 M), and 1-phenylethanol (0.5 M), respectively, with *tert*-butyl hydroperoxide (1.5 M) catalyzed by complex **2** (5 × 10^−4^ M) at 50 °C in acetonitrile. Accumulation of acetophenone (graph **C**, curve 2) in the oxidation of 1-phenylethanol (0.5 M) with H_2_O_2_ (2,0 M) catalyzed by complex **2** (5 × 10^−4^ M) at 50 °C in acetonitrile. To quench the oxidation process, concentrations of products were measured by GC only after reducing the reaction sample with solid PPh_3_.

Figure 12A–C represent kinetic curves of ketone accumulation during the oxidation of alcohols (including three substrates, 2-heptanol, cyclohexanol, and 1-phenylethanol) with *tert*-butylhydroperoxide in acetonitrile. 1-Phenylethanol is the most active substrate. It should be noted that the oxidation with hydrogen peroxide of this alcohol proceeds much less efficiently (see curve 2 in Figure 12C). Attention should be drawn to the fact that the oxidation of phenylethanol with hydrogen peroxide is low compared to *tert*-butyl hydroperoxide (Figure 12C). In contrast, the alkane oxidation with hydrogen peroxide in the presence of the same catalyst is extremely efficient under the same conditions (Figure 7). Perhaps, the mechanism of decomposition of hydrogen peroxide and the rate of the generation of particles induces the oxidation of alcohol change in the presence of alcohol. To establish the reason for this puzzling observation, more studies are needed in the future.

## 3. Conclusions

The very first examples of metallacomplexes, including simultaneously silsesquioxane and 8-hydroxyquinoline-derived ligands, were prepared via a convenient self-assembly approach. The exchange reaction of alkali-metal-based (M = Li, Na, or K) phenylsiloxanolates [PhSi(O)OM]_x_ and copper (II) chloride, followed by the complexation with ligands of the 8-hydroxyquinoline family, smoothly lead to a series of similar sandwich-like octanuclear (Cu_6_M_2_) cage-like complexes. Each cage structure includes two pairs of ligands: two pentameric (Ph_5_Si_5_O_10_) cycles and two N,O-ligands of the 8-hydroxyquinolinolate type (derived from 8-hydroxyquinoline, 5-chloro-8-hydroxyquinoline, 5,7-dibromo-8-hydroxyquinoline, or 5,7-diiodo-8-hydroxyquinoline). Hydroxyl groups of such N,O-ligands are metallated by the corresponding metal ions (lithium, sodium, or potassium). The size of these metal ions is responsible for the supramolecular packing of cage complexes: (*i*) lithium-based compounds are island-like structures, (*ii*) potassium-based compounds are coordination polymers, and (*iii*) “intermediate” sodium-based compounds could realize both options, depending on additional factors. In sum, the use of a single type of organic ligand of the 8-hydroxyquinoline family allowed us to develop an impressively powerful approach to similar (in terms of the molecular cage structure) complexes, exhibiting principally different features of supramolecular packing (from discrete 0D to extended 3D geometry). The representative complex Cu_6_Na_2_-silsesquioxane/8-hydroxyquinoline **2** exhibits high catalytic activity in the oxidation of hydrocarbons and alcohols with H_2_O_2_ or *tert*-butyl hydroperoxide. Studies of kinetics and selectivity led us to the conclusion that the oxidation proceeds mainly with the participation of free hydroxyl radicals, as evidenced by the specific regioselectivity for individual chemical bonds in the oxidation of *n*-heptane and methylcyclohexane, as well as the dependence of the reaction rate on the initial concentration of cyclohexane. Of particular importance is the addition of nitric acid to hydrogen peroxide as a co-catalyst. Both research lines, (*i*) the design of heteroligand metallasilsesquioxane complexes and (*ii*) their evaluation in catalytic applications, are currently in further progress in our groups.

## Supplementary Materials

The following supporting information can be downloaded at: https://www.mdpi.com/article/10.3390/molecules27196205/s1: Details of syntheses (including isolation and T-stacking structure of complex **3a**), catalyzed reactions, and X-ray diffraction studies (CCDC 2203816-2203826) [105,106,107,108,109,110,111,112,113,114].

## Sample Availability

Samples of the compounds are not available from the authors.
