# Supplementary material for: A Novel Family of Cage-like (CuLi, CuNa, CuK)-phenylsilsesquioxane Complexes with 8-Hydroxyquinoline Ligands: Synthesis, Structure, and Catalytic Activity
Source: Molecules. 2022 Sep 21;27(19):6205. doi: 10.3390/molecules27196205 (PMC9571593; doi:10.3390/molecules27196205)
Supplement: Supplementary file 1 [file molecules-27-06205-s001.zip › molecules-1914449-supplementary.pdf]

Family of cagelike (CuLi, CuNa, CuK)-phenylsilsesquioxane complexes with 8-hydroxyquinoline ligands: synthesis, structure, catalytic activity

Alexey N. Bilyachenko,*[1,2] Victor N. Khrustalev,[2,3] Anna Y. Zueva,[1,2] Grigorii S. Astakhov,[1,2] Ekaterina M. Titova,[1] Yan V. Zubavichus,[4] Pavel V. Dorovatovskii,[5] Alexander A. Korlyukov,[1,6] Lidia S. Shul'pina,[1] Elena S. Shubina,[1] Yuriy N. Kozlov,[7,8] Nikolay S. Ikonnikov,[1] Dmitri Gelman,[9] and Georgiy B. Shul'pin*[6,7]

[1]A.N. Nesmeyanov Institute of Organoelement Compounds, Russian Academy of Sciences, Vavilov Str. 28, Moscow, 119991, Russia

[2]Peoples' Friendship University of Russia (RUDN University), Miklukho-Maklay Str. 6, Moscow, Russia

[3]Zelinsky Institute of Organic Chemistry, Russian Academy of Sciences, Leninsky Prospect 47, Moscow 119991, Russia

[4]Synchrotron Radiation Facility SKIF, Boreskov Institute of Catalysis SB RAS, Nikolskii prosp., 1, Koltsovo 630559, Russia

[5]National Research Center "Kurchatov Institute", pl. Akad. Kurchatova 1, Moscow, 123182, Russia

[6]Pirogov Russian National Research Medical University, Ostrovitianov Str., 1, Moscow 117997, Russia

[7]Semenov Federal Research Center for Chemical Physics, Russian Academy of Sciences, ul. Kosygina 4, Moscow 119991, Russia

[8]Plekhanov Russian University of Economics, Academic Department of Innovational Materials and Technologies Chemistry, Stremyanny pereulok 36, Moscow, 117997, Russia

[9]Institute of Chemistry, Edmond J. Safra Campus, The Hebrew University of Jerusalem, 91904 Jerusalem, Israel

General experimental considerations

All reagents were purchased from the usual suppliers (Sigma, Fluka) and used without further purification. Elemental analyses were carried out with an XRF spectrometer VRA-30. IR spectra of the compounds (KBr pellets) were measured on a Shimadzu IR Prestige 21 FT-IR Spectrophotometer equipped with an MCT detector using a Miracle single reflection ATR unit by Pike. Set of signals: 1600-1400 cm^{-1} (νC=C, νC=N), 1120 cm^{-1} (νPh–Si), 940–1100 cm^{-1} (ν_{as}Si–O, ν_{as}Si–O–Si), 900 cm^{-1} (ν_{as}Si–O in Si–O–M fragment), 720–680 cm^{-1} (σC–H of mono-substituted phenyl group). Figure S2 is to exemplify a general view of spectra (see below). UV-Vis spectra (10 mm optical path length, ethanol solutions) were recorded on a Cary 50 spectrophotometer. Figure S3 is to exemplify a general view of spectra (see below).

Scheme S1. General scheme of synthesis of CuNa-silsesquioxane complex with 5-chloro-8-hydroxyquinoline ligands **3a**. Solvated molecules are omitted for clarity

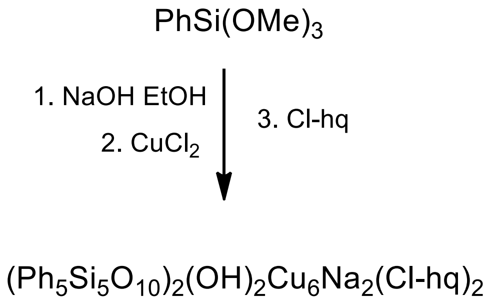

3a

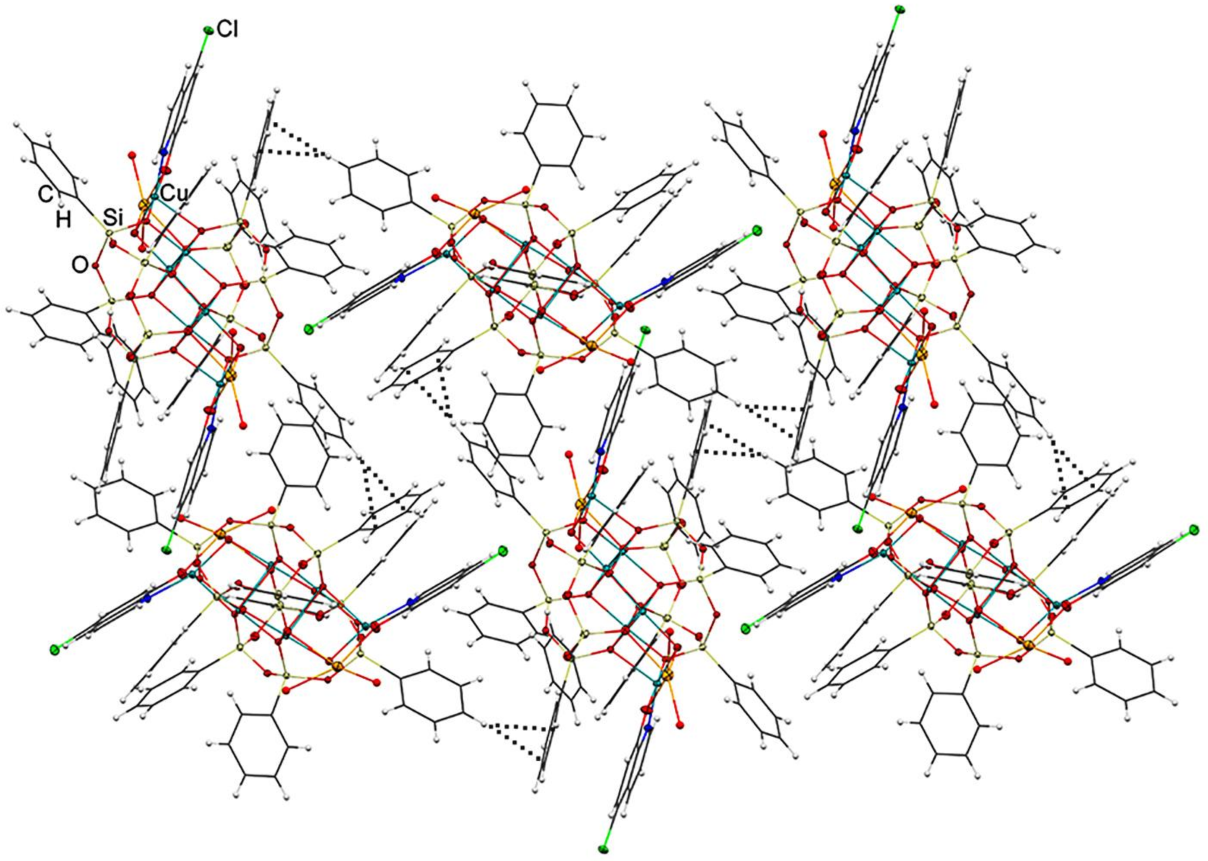

Figure S1. T-stacking interactions between phenyl groups at neighboring cages in crystal packing of complex **3a**

Syntheses of **1**, **1a**, **6**.

In a typical procedure, 1.50 g (7.56 mmol) of $PhSi(OMe)_3$ and 0.25 g (10 mmol) of LiOH were heated at reflux in 40 ml of ethanol for 1.5 h. Then, 0.61 g (4.54 mmol) of $CuCl_2$ was added and the resulting mixture was stirred without heating for 24 h). Afterwards, 0.22 g (1.51 mmol) of 8-hydroxyquinoline (0.46 g (1.51 mmol) of 5,7-dibromo-8-hydroxyquinoline in case of **6**) was added and the resulting

mixture was stirred without heating for 3 h followed by the centrifugation of precipitate. Crystallization of filtrates gave in 3-4 days a crystalline material, including single crystals that were used for X-ray diffraction analysis. The remaining part of the crystalline material was dried in vacuum to calculate yields (see below for details).

Complex **1**. Anal. Calcd for $(Ph_5Si_5O_{10})_2(OH)_2Cu_6Li_2(C_9H_7NO)_2$: Cu, 18.23; Li, 0.66; N, 1.34; Si, 13.43. Found: Cu, 18.17; Li, 0.61; N, 1.30; Si, 13.35. Yield: 0.52 g (33%).

Complex **1a**. Anal. Calcd for $(Ph_5Si_5O_{10})_2(OH)_2Cu_6Li_2(C_9H_7NO)_2$: Cu, 18.23; Li, 0.66; N, 1.34; Si, 13.43. Found: Cu, 18.18; Li, 0.64; N, 1.29; Si, 13.40. Yield: 0.61 g (39%).

Complex **6**. Anal. Calcd for $(Ph_5Si_5O_{10})_2(OH)_2Cu_6Li_2(C_9H_5Br_2NO)_2$: Br, 13.28; Cu, 15.84; Li, 0.58; N, 1.16; Si, 11.67. Found: Br, 13.20; Cu, 15.79; Li, 0.55; N, 1.12; Si, 11.61. Yield: 0.32 g (18%).

Syntheses of **2**, **3**, **3a**, **4**, **7**.

In a typical procedure, 1.50 g (7.56 mmol) of $PhSi(OMe)_3$ and 0.42 g (10 mmol) of NaOH were heated at reflux in 40 ml of ethanol for 1.5 h. Then, 0.61 g (4.54 mmol) of $CuCl_2$ was added and the resulting mixture was stirred without heating for 24 h). Afterwards, 0.22 g (1.51 mmol) of 8-hydroxyquinoline (0.27 g (1.51 mmol) 5-chloro-8-hydroxyquinoline in case of **3**; 0.46 g (1.51 mmol) of 5,7-dibromo-8-hydroxyquinoline in case of **4** and **7**) was added and the resulting mixture was stirred without heating for 3 h followed by the centrifugation of precipitate. Filtrate of **3** was mixed with 15 ml of butanol. Filtrate of **4** was mixed with 15 ml of acetone. Crystallization of filtrates gave in 3-4 days a crystalline material, including single crystals that were used for X-ray diffraction analysis. The remaining part of the crystalline material was dried in vacuum to calculate yields (see below for details).

Complex **2**. Anal. Calcd for $(Ph_5Si_5O_{10})_2(OH)_2Cu_6Na_2(C_9H_7NO)_2$: Cu, 17.96; N, 1.32; Na, 2.17; Si, 13.23. Found: Cu, 17.90; N, 1.27; Na, 2.11; Si, 13.19. Yield: 0.51 g (32%).

Complex **3**. Anal. Calcd for $(Ph_5Si_5O_{10})_2(OH)_2Cu_6Na_2(C_9H_6ClNO)_2$: Cl, 3.23; Cu, 17.39; N, 1.28; Na, 2.10; Si, 12.81. Found: Cl, 3.19; Cu, 17.32; N, 1.22; Na, 2.07; Si, 12.77. Yield: 0.33 g (20%).

Complex **3a**. Anal. Calcd for $(Ph_5Si_5O_{10})_2(OH)_2Cu_6Na_2(C_9H_6ClNO)_2$: Cl, 3.23; Cu, 17.39; N, 1.28; Na, 2.10; Si, 12.81. Found: Cl, 3.19; Cu, 17.32; N, 1.22; Na, 2.07; Si, 12.77. Yield: 0.42 g (26%).

Complex **4**. Anal. Calcd for $(Ph_5Si_5O_{10})_2(OH)_2Cu_6Na_2(C_9H_5Br_2NO)_2$: Br, 13.10; Cu, 15.63; N, 1.15; Na, 1.89; Si, 11.51. Found: Br, 13.03; Cu, 15.59; N, 1.11; Na, 1.84; Si, 11.47. Yield: 0.81 g (44%).

Complex **7**. Anal. Calcd for $(Ph_5Si_5O_{10})_2(OH)_2Cu_6Na_2(C_9H_5Br_2NO)_2$: Br, 13.10; Cu, 15.63; N, 1.15; Na, 1.89; Si, 11.51. Found: Br, 13.00; Cu, 15.57; N, 1.12; Na, 1.86; Si, 11.45. Yield: 0.49 g (27%).

Syntheses of **5**, **8**, **9**

In a typical procedure, 1.50 g (7.56 mmol) of $PhSi(OMe)_3$ and 0.59 g (10 mmol) of KOH were heated at reflux in 40 ml of ethanol (methanol in case of **9**) for 1.5 h. Then, 0.61 g (4.54 mmol) of $CuCl_2$ was added and the resulting mixture was stirred without heating for 24 h). Afterwards, 0.22 g (1.51 mmol) of 8-hydroxyquinoline (0.46 g (1.51 mmol) of 5,7-dibromo-8-hydroxyquinoline in case of **8**, 0.6 g (1.51 mmol) of 5,7-iodo-8-hydroxyquinoline in case of **9**) was added and the resulting mixture was stirred without heating for 3 h followed by the centrifugation of precipitate. Filtrate of **8** was mixed with 15 ml of acetone. Crystallization of filtrates gave in 3-4 days a crystalline material, including single crystals that were used for X-ray diffraction analysis. The remaining part of the crystalline material was dried in vacuum to calculate yields (see below for details).

Complex **5**. Anal. Calcd for $(Ph_5Si_5O_{10})_2(OH)_2Cu_6K_2(C_9H_7NO)_2$: Cu, 17.69; K, 3.63; N, 1.30; Si, 13.03. Found: Cu, 17.61; K, 3.57; N, 1.25; Si, 12.94. Yield: 0.60 g (37%).

Complex **8**. Anal. Calcd for $(Ph_5Si_5O_{10})_2(OH)_2Cu_6K_2(C_9H_5Br_2NO)_2$: Br, 12.93; Cu, 15.43; K, 3.16; N, 1.13; Si, 11.36. Found: Br, 12.90; Cu, 15.38; K, 3.12; N, 1.09; Si, 11.30. Yield: 0.73 g (39%).

Complex **9**. Anal. Calcd for $(Ph_5Si_5O_{10})_2(OH)_2Cu_6K_2(C_9H_5I_2NO)_2$: Cu, 14.34; I, 19.09; K, 2.94; N, 1.05; Si, 10.56. Found: Cu, 14.29; I, 19.02; K, 2.90; N, 1.01; Si, 10.51. Yield: 0.66 g (33%).

The oxidation of alkanes with hydrogen peroxide was typically carried out in air in thermostated Pyrex cylindrical vessels with vigorous stirring. Total volume of the reaction solution was 5 mL (**CAUTION**: the combination of air or molecular oxygen and H_2O_2 with organic compounds at elevated temperatures may be explosive!). Initially, a portion of 50% aqueous solution of hydrogen peroxide was added to the solution of the catalyst and substrate in acetonitrile. The aliquots of the reaction solution were analysed by GC (the instrument 3700, fused silica capillary column FFAP/OV-101 20/80 w/w, 30 m × 0.2 mm × 0.3 µm; helium as a carrier gas. Attribution of peaks was made by comparison with chromatograms of authentic samples. Usually samples were analyzed twice, i.e. before and after the addition of the excess of solid PPh_3. This method was developed and used previously by Shul'pin [57, S1-S3]. Alkyl hydroperoxides are transformed in the GC injector into a mixture of the corresponding ketone and alcohol. Due to this we quantitatively reduced the reaction samples with PPh_3 to obtain the corresponding alcohol. This method allows us to calculate the real concentrations not only of the hydroperoxide but of the alcohols and ketones present in the solution at a given moment.

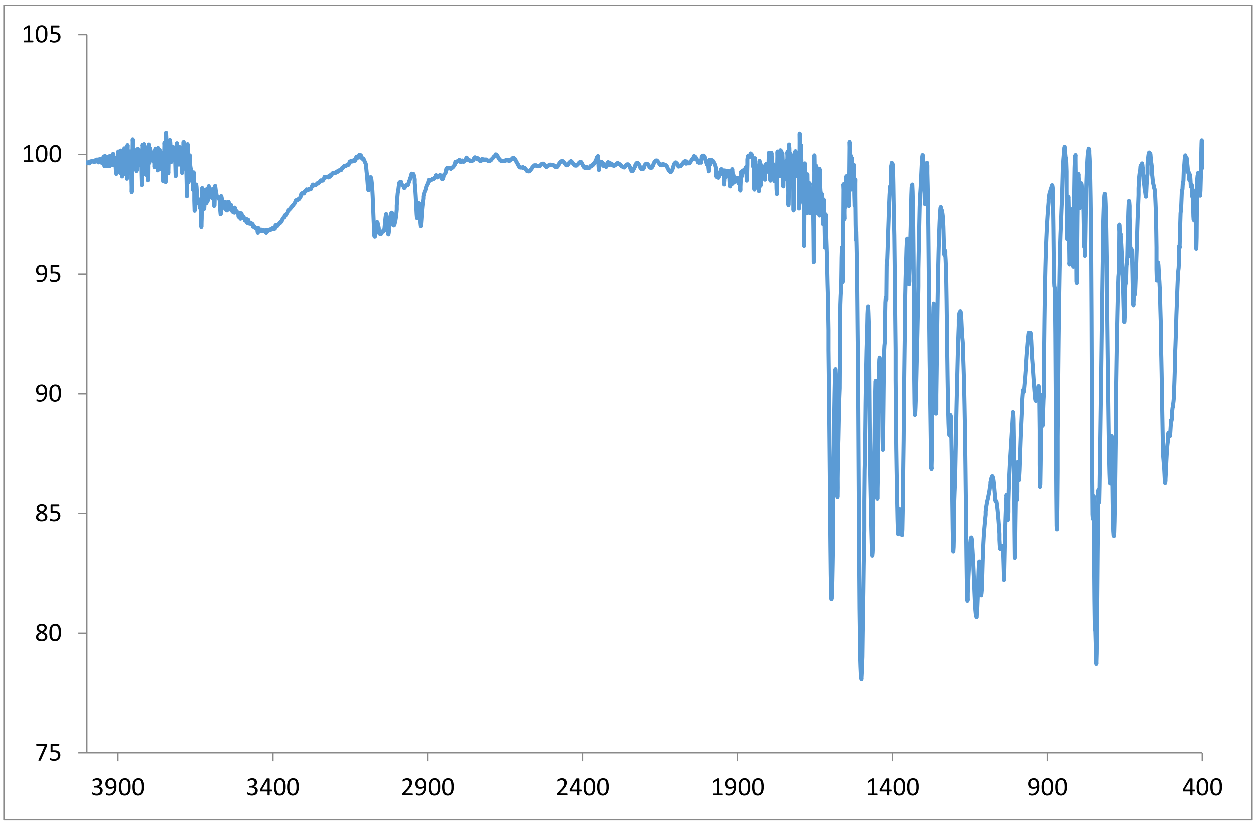

Figure S2. IR spectrum of **2**

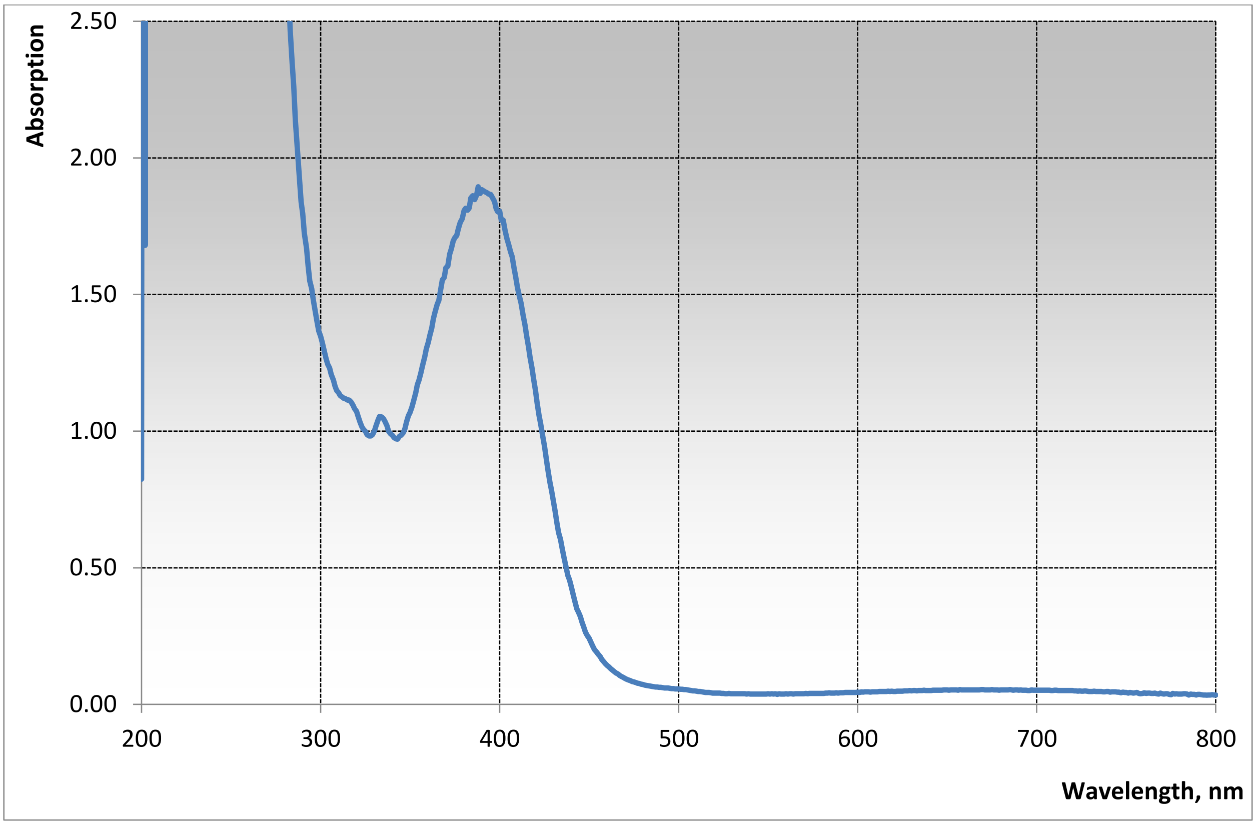

Figure S3. UV-vis spectrum of **2**.

X-ray crystal structure determination

X-ray diffraction data were collected on a three-circle Bruker D8 QUEST PHOTON-III CCD diffractometer (graphite monochromator, φ and ω scan mode) (**1**), a four-circle Rigaku Synergy S diffractometer equipped with a HyPix6000HE area-detector (kappa geometry, shutterless φ and ω-scan mode) (**1a**, **6-9**), a three-circle Bruker SMART 1K CCD diffractometer (graphite monochromator, φ and ω scan mode) (**2**, **3**, **3a**) and on the 'Belok' beamline of the National Research Center 'Kurchatov Institute' (oscillation range of 1.0°, φ scan mode) using a Rayonix SX165 CCD detector (**4**, **5**). For **1**, **2**, **3** and **3a**, the data were integrated by the SAINT program [S4] and corrected for absorption by the SADABS program [S5]. For **1a**, **6-9**, the data were integrated and corrected for absorption by the CrysAlisPro program [S6]. For **4** and **5**, the data were integrated by the utility *iMOSFLM* in CCP4 program [S7] and corrected for absorption by the *Scala* program [S8]. The structures were determined by direct methods and refined by full-matrix least squares technique on F^2 with anisotropic displacement parameters for non-hydrogen atoms. For details, see Table S1. All attempts to model and refine positions of the ethanol (in **1a**) and several acetone (in **8**) solvate molecules were unsuccessful. Therefore, their contribution to the total scattering pattern was removed by use of the utility *SQUEEZE* in PLATON15 [S9]. The hydrogen atoms of the OH-groups were localized in the difference-Fourier map and included in the refinement within the riding model with fixed isotropic displacement parameters. The other hydrogen atoms were placed in calculated positions and refined within riding model with fixed isotropic displacement parameters [U_{iso}(H) = 1.5U_{eq}(C) for the CH_3-groups and 1.2U_{eq}(C) for the other groups]. All calculations were carried out using the SHELXTL program [S10].
Crystallographic data for **1**, **1a**, **2**, **3**, **3a** and **4-9** have been deposited with the Cambridge Crystallographic Data Center, CCDC 2203816-2203826, respectively. Copies of this information may be obtained free of charge from the Director, CCDC, 12 Union Road, Cambridge CB2 1EZ, UK (Fax: +44 1223 336033; e-mail: deposit@ccdc.cam.ac.uk or www.ccdc.cam.ac.uk).

Table S1. Crystal data and structure refinement for complexes **1**, **1a**, **2**, **3**, **3a**.

Identification code	**1** • EtOH	**1a** • *2EtOH*$_{sq}$	**2** • EtOH	**3**	**3a**
Empirical formula	$C_{84}H_{82}Li_2N_2O_{27}Si_{10}Cu_6$	$C_{81.2}H_{74.4}Li_2N_2O_{26}Si_{10}Cu_6$	$C_{86}H_{90}N_2Na_2O_{29}Si_{10}Cu_6$	$C_{94}H_{102}N_2O_{28}Na_2Si_{10}Cl_2Cu_6$	$C_{86}H_{86}N_2O_{28}Na_2Si_{10}Cl_2Cu_6$
Formula weight	2227.60	2170.31	2323.79	2486.85	2374.58
Temperature, K	100(2)	100(2)	296(2)	293(2)	120(2)
Crystal size, mm	0.20 × 0.18 × 0.12	0.20 × 0.15 × 0.12	0.36 × 0.03 × 0.03	0.23 × 0.18 × 0.16	0.35 × 0.31 × 0.23
Wavelength, Å	0.71073	1.54184	0.71073	0.71073	0.71073
Crystal system	Monoclinic	Monoclinic	Monoclinic	Monoclinic	Monoclinic
Space group	$P2_1$/n	$P2_1$/n	$P2_1$/n	$C2$/c	$P2_1$/n
a, Å	17.4064(7)	17.4793(4)	17.652(9)	35.071(5)	18.4547(7)
b, Å	16.5555(7)	16.5679(3)	17.772(9)	16.183(2)	16.3798(6)
c, Å	19.1296(8)	19.1838(4)	18.409(9)	21.800(3)	19.7528(7)
α, deg.	90	90	90	90	90
β, deg.	112.689(1)	112.801(2)	113.176(7)	92.701(3)	111.358(1)
γ, deg.	90	90	90	90	90
V, Å^3	5086.0(4)	5121.4(2)	5309(5)	12359(3)	5560.9(4)
Z	2	2	2	4	2
Density (calc.), mg/m^3	1.455	1.407	1.454	1.336	1.418
μ, mm^{-1}	1.420	3.051	1.373	1.225	1.358
F(000)	2272	2207	2376	5096	2420
Theta range, deg.	2.62 to 27.53	2.91 to 78.05	1.70 to 26.00	1.66 to 30.65	1.67 to 26.37
Index ranges	$-22 \le h \le 22$,<br>$-21 \le k \le 21$,<br>$-24 \le l \le 24$	$-22 \le h \le 22$,<br>$-20 \le k \le 18$,<br>$-24 \le l \le 24$	$-21 \le h \le 21$,<br>$-21 \le k \le 21$,<br>$-22 \le l \le 22$	$-50 \le h \le 50$,<br>$-23 \le k \le 23$,<br>$-30 \le l \le 31$	$-23 \le h \le 22$,<br>$-20 \le k \le 20$,<br>$-24 \le l \le 24$
Reflections collected	82192	89678	37859	83833	50218
Independent reflections	11588 (R_{int} = 0.0494)	10805 (R_{int} = 0.0501)	10177 (R_{int} = 0.1704)	18770 (R_{int} = 0.0864)	11345 (R_{int} = 0.0294)
Reflections observed	8217	9127	4997	11431	9032
Restraints / parameters	183 / 475	169 / 517	9 / 526	56 / 688	108 / 641
R_1 / wR_2 ($I > 2\sigma(I)$)	0.0784 / 0.1827	0.0713 / 0.1784	0.1503 / 0.2966	0.0621 / 0.1518	0.0537 / 0.1501
R_1 / wR_2 (all data)	0.1025 / 0.2001	0.0808 / 0.1878	0.2601 / 0.3460	0.1191 / 0.1886	0.0706 / 0.1691
Goodness-of-fit on F^2	1.081	1.001	1.070	1.010	1.044
Extinction coefficient	—	—	—	—	—
T_{min} / T_{max}	0.752 / 0.835	0.531 / 0.660	0.606 / 0.927	0.663 / 0.817	0.625 / 0.746
$\Delta\rho_{max}$ / $\Delta\rho_{min}$, e·Å^{-3}	1.480 / -0.963	1.243 / -0.817	2.243 / -1.319	1.555 / -0.800	1.981 / -1.110

Table S2. Crystal data and structure refinement for complexes **4-8**.

Identification code	**4** • $2Me_2CO$ • EtOH	**5**	**6** • 4EtOH	**7** • 4EtOH	**8** • $2Me_2CO$ • *$2Me_2CO_{sq}$*
Empirical formula	$C_{93}H_{98}N_2O_{31}Na_2Si_{10}Cu_6Br_4$	$C_{82}H_{76}N_2O_{26}Si_{10}K_2Cu_6$	$C_{90}H_{96}Li_2N_2O_{30}Si_{10}Cu_6Br_4$	$C_{90}H_{96}N_2O_{30}Na_2Si_{10}Cu_6Br_4$	$C_{96}H_{96}N_2O_{30}Si_{10}K_2Cu_6Br_4$
Formula weight	2767.52	2245.85	2681.37	2713.47	2817.76
Temperature, K	100(2)	100(2)	100(2)	100(2)	100(2)
Crystal size, mm	0.06 × 0.03 × 0.01	0.14 × 0.11 × 0.09	0.16 × 0.10 × 0.09	0.27 × 0.23 × 0.16	0.27 × 0.18 × 0.16
Wavelength, Å	Synchrotron (λ = 0.79313)	Synchrotron (λ = 0.79313)	1.54184	1.54184	1.54184
Crystal system	Monoclinic	Monoclinic	Monoclinic	Monoclinic	Monoclinic
Space group	$P2_1/n$	$P2_1/n$	$P2_1/n$	$P2_1/n$	$P2_1/n$
a, Å	18.9311(17)	15.4020(13)	13.2691(7)	13.90223(13)	15.68582(12)
b, Å	16.4169(15)	20.1680(18)	24.0845(11)	23.3619(3)	23.8523(2)
c, Å	19.6530(18)	15.7450(14)	16.3473(9)	16.2719(2)	15.89768(13)
α, deg.	90	90	90	90	90
β, deg.	111.702(11)	111.188(9)	93.099(5)	91.8573(12)	99.8697(8)
γ, deg.	90	90	90	90	90
V, Å^3	5675.0(10)	4560.2(7)	5216.6(5)	5282.05(11)	5859.98(8)
Z	2	2	2	2	2
Density (calc.), mg/m^3	1.620	1.636	1.707	1.706	1.597
μ, mm^{-1}	3.582	2.255	4.883	4.908	5.006
F(000)	2788	2284	2700	2732	2836
Theta range, deg.	2.49 to 31.00	2.10 to 30.99	3.81 to 84.93	3.31 to 78.17	3.38 to 77.84
Index ranges	$-24 \le h \le 24$, $-19 \le k \le 21$, $-25 \le l \le 25$	$-19 \le h \le 19$, $-26 \le k \le 26$, $-20 \le l \le 20$	$-16 \le h \le 16$, $-30 \le k \le 21$, $-20 \le l \le 20$	$-17 \le h \le 16$, $-29 \le k \le 28$, $-20 \le l \le 20$	$-19 \le h \le 19$, $-29 \le k \le 30$, $-20 \le l \le 19$
Reflections collected	41587	46466	62801	69228	76985
Independent reflections	12796 (R_{int} = 0.0754)	10278 (R_{int} = 0.0443)	10871 (R_{int} = 0.0798)	11101 (R_{int} = 0.0781)	12297 (R_{int} = 0.0461)
Reflections observed	8170	7120	7282	8657	10538
Restraints / parameters	19 / 650	0 / 579	9 / 616	12 / 632	0 / 676
R_1 / wR_2 ($I > 2\sigma(I)$)	0.0912 / 0.2155	0.0470 / 0.1203	0.0747 / 0.1893	0.0682 / 0.1729	0.0499 / 0.1317
R_1 / wR_2 (all data)	0.1346 / 0.2436	0.0767 / 0.1405	0.1071 / 0.2226	0.0866 / 0.1992	0.0583 / 0.1419
Goodness-of-fit on F^2	1.052	1.017	1.030	1.017	1.023
Extinction coefficient	0.0010(1)	0.00058(6)	—	—	—
T_{min} / T_{max}	0.781 / 0.945	0.721 / 0.802	0.454 / 0.623	0.268 / 0.440	0.191 / 0.500
$\Delta\rho_{max}$ / $\Delta\rho_{min}$, e·Å^{-3}	2.104 / -1.049	0.628 / -0.562	1.127 / -0.837	1.175 / -1.220	1.214 / -0.764

Table S3. Crystal data and structure refinement for complex **9**.

Identification code	**9** • ⅙MeOH
Empirical formula	$C_{117.25}H_{91}N_3O_{36.25}Si_{15}K_3Cu_9I_6$
Formula weight	3993.93
Temperature, K	100(2)
Crystal size, mm	0.11 × 0.09 × 0.06
Wavelength, Å	1.54184
Crystal system	Trigonal
Space group	*R*-3
a, Å	37.6662(5)
b, Å	37.6662(5)
c, Å	16.8708(3)
α, deg.	90
β, deg.	90
γ, deg.	120
V, $Å^3$	20728.6(7)
Z	6
Density (calc.), mg/m^3	1.920
μ, mm^{-1}	14.714
F(000)	11709
Theta range, deg.	2.35 to 77.99
Index ranges	$-47 \leq h \leq 41$,<br>$-47 \leq k \leq 47$,<br>$-21 \leq l \leq 21$
Reflections collected	172555
Independent reflections	9805 (R_{int} = 0.1036)
Reflections observed	7273
Restraints / parameters	32 / 568
R_1 / wR_2 ($I > 2\sigma(I)$)	0.0665 / 0.1754
R_1 / wR_2 (all data)	0.0881 / 0.1991
Goodness-of-fit on F^2	1.041
Extinction coefficient	—
T_{min} / T_{max}	0.234 / 0.400
$\Delta\rho_{max}$ / $\Delta\rho_{min}$, e·$Å^{-3}$	1.894 / -1.172

S1. G. B. Shul'pin. Metal-catalysed hydrocarbon oxygenations in solutions: the dramatic role of additives: a review. *J. Mol. Catal., A: Chem.* **2002**, *189*, 39–66, https://doi.org/10.1016/S1381-1169(02)00196-6

S2. Olivo, G.; Lanzalunga, O.; Di Stefano, S. Non-Heme Imine-Based Iron Complexes as Catalysts for Oxidative Processes (Review). *Adv. Synth. Catal.* **2016**, *358*, 843–863, https://doi.org/10.1002/adsc.201501024

S3. Czerwińska, K.; Machura, B.; Kula, S.; Krompiec, S.; Erfurt, K.; Roma-Rodrigues, C.; Fernandes, A. R.; Shul'pina, L. S.; Ikonnikov, N. S.; Shul'pin, G. B. Copper(II) complexes of functionalized 2,2':6',2''-terpyridines and 2,6-di(thiazol-2-yl)pyridine: structure, spectroscopy, cytotoxicity and catalytic activity. *Dalton Trans.* **2017**, *46*, 9591-9604, https://doi.org/10.1039/C7DT01244F

S4. Bruker, *SAINT*, Bruker AXS Inc., Madison, WI, **2013**.

S5. Krause, L., Herbst-Irmer, R., Sheldrick G.M., Stalke D., Comparison of silver and molybdenum microfocus X-ray sources for single-crystal structure determination. *J. Appl. Cryst.* **2015**, *48*, 3-10.

S6. CrysAlisPro, Version 1.171.41.106a. *Rigaku Oxford Diffraction*, **2021**.

S7. T. G. G. Battye, L. Kontogiannis, O. Johnson, H. R. Powell, A. G. W. Leslie, *iMOSFLM*: a new graphical interface for diffraction-image processing with *MOSFLM*. *Acta Cryst.* **2011**, *D67*, 271-281.

S8. P. R. Evans, Scaling and assessment of data quality. *Acta Cryst.* **2006**, *D62*, 72-82.

S9. A. L. Spek, *PLATON*, A Multipurpose Crystallographic Tool, Utrecht University, the Netherlands, **2015**.

S10. Sheldrick, G. M. Crystal structure refinement with *SHELXL*. *Acta Cryst.* **2015**, *C71*, 3-8.
